# Supplementary material for: The interactome of intact mitochondria by cross-linking mass spectrometry provides evidence for coexisting respiratory supercomplexes
Source: Mol Cell Proteomics. 2017 Dec 8;17(2):216–32. doi: 10.1074/mcp.RA117.000470 (PMC5795388; doi:10.1074/mcp.RA117.000470)
Supplement: Supplemental Data [file supp_RA117.000470_133922_0_supp_23978_fzffwf.zip › spectra_annotation/mito_spectra_annotation/147-1-14-1-12-1.pdf]

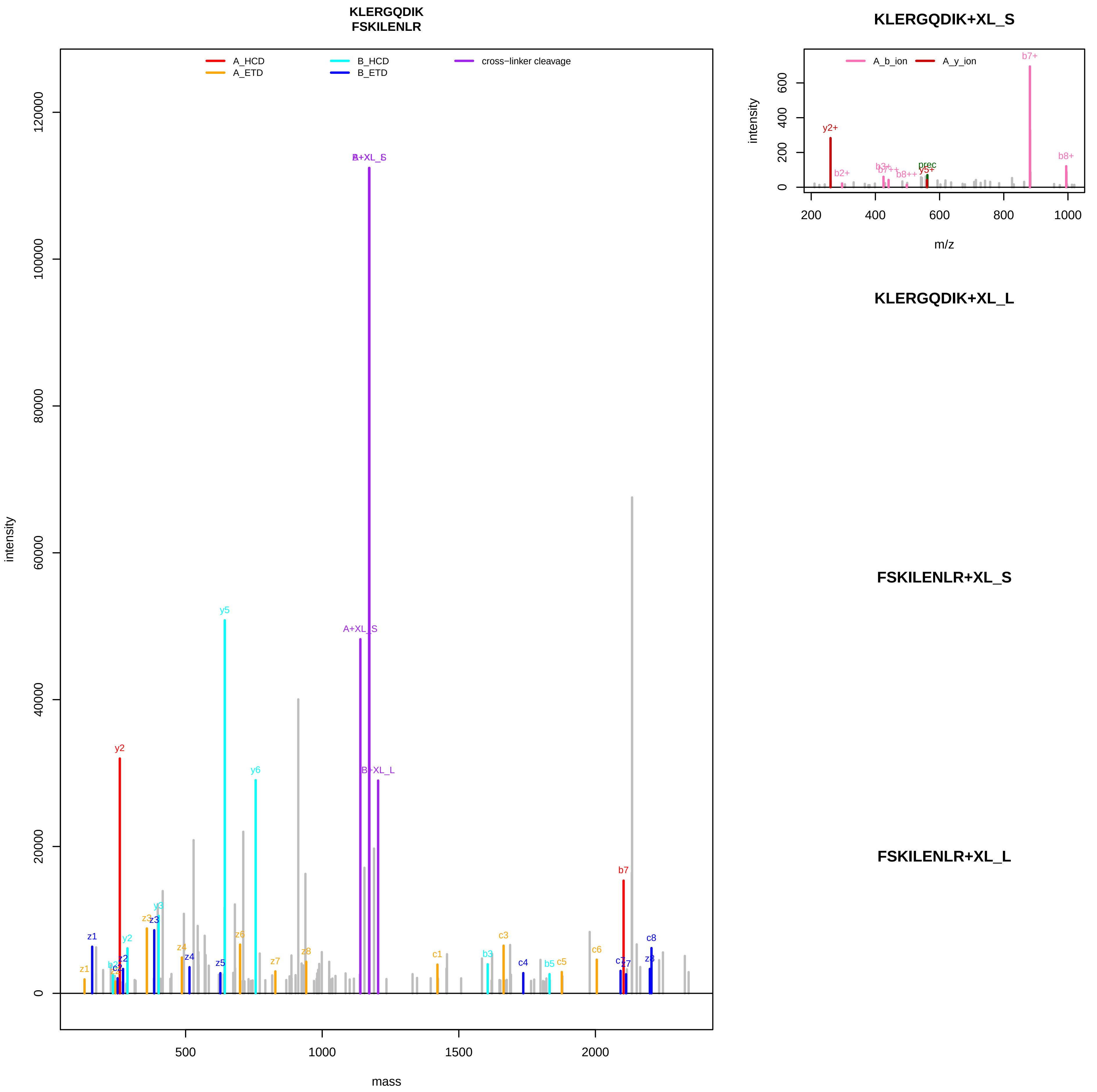
KLERGQDIK
FSKILENLR
A_HCD
A_ETD
B_HCD
B_ETD
cross−linker cleavage
intensity
mass
0
20000
40000
60000
80000
100000
120000
500
1000
1500
2000
A+XL_S
B+XL_S
B+XL_L
y2
y5
y6
b7
c1
c3
c5
c6
c7
c8
z1
z3
z4
z5
z6
z7
z8
b3
b5
KLERGQDIK+XL_S
A_b_ion
A_y_ion
intensity
m/z
0
200
400
600
800
1000
y2+
b2+
b3+
b7++
b8++
prec
y5+
b7+
b8+
KLERGQDIK+XL_L
FSKILENLR+XL_S
FSKILENLR+XL_L
